# The Loss of miR-26a-Mediated Post-Transcriptional Regulation of Cyclin E2 in Pancreatic Cancer Cell Proliferation and Decreased Patient Survival

**DOI:** 10.1371/journal.pone.0076450

**Published:** 2013-10-08

**Authors:** Jingjing Deng, Miaoxia He, Lizao Chen, Chao Chen, Jianming Zheng, Zailong Cai

**Affiliations:** 1 Department of Pathology, Changhai Hospital, Second Military Medical University, Shanghai, China; 2 Department of Biochemistry and Molecular Biology, Second Military Medical University, Shanghai, China; Ohio State University Comprehensive Cancer Center, United States of America

## Abstract

**Background:**

miR-26a plays a critical role in tumorigenesis, either as a tumor suppressor or as an oncogenic miRNA, depending on different tumor types. However, the function of miR-26a in pancreatic cancer has not been clearly elucidated. The present study was designed to determine the roles of miR-26a in pancreatic cancer and its association with the survival of patients with pancreatic cancer.

**Methods:**

The expression of miR-26a was examined in 15 pairs of pancreatic duct adenocarcinoma (PDAC) and their adjacent benign pancreatic tissues (ABPT), by qRT-PCR. The results were confirmed by *in situ* hybridization using two panels of 106 PDACs and their ABPT microarray. The association of miR-26a expression with overall survival was determined. The proliferation and cell cycle distribution of Capan-2, SW-1990, and Panc-1 cells, transfected with miR-26a mimics or a miR-26a inhibitor, were assessed using the Cell Counting Kit-8 assay and flow cytometry, respectively. The cell tumorigenicity was evaluated via murine xenograft experiments. Cyclin D2, E2, EZH2, and PCNA levels were analyzed by Western blot and immunohistochemistry.

**Results:**

miR-26a was expressed in the cytoplasm of pancreatic ductal epithelial cells, whereas its expression was significantly downregulated in PDAC tissues compared with that of ABPT. Patients with low miR-26a expression had a significantly shorter survival than those with high miR-26a expression. The *in vitro* and *in vivo* assays showed that overexpression of miR-26a resulted in cell cycle arrest, inhibited cell proliferation, and decreased tumor growth, which was associated with cyclin E2 downregulation.

**Conclusions:**

miR-26a is an important suppressor of pancreatic ductal carcinoma, and can prove to be a novel prognostic factor and therapeutic target for pancreatic cancer treatment.

## Introduction

Pancreatic cancer, particularly pancreatic duct adenocarcinoma (PDAC), is the fourth most common cause of cancer-related deaths worldwide. With a median survival time of less than 6 months and an average 5-year survival rate of less than 5%, the mortality–incidence ratio for patients with pancreatic cancer is approximately 99%, with extremely poor prognosis [1], [2]. Therefore, molecular mechanisms involved in the tumor malignant transformation process, including the role of microRNAs (miRNAs), must be understood for the improved diagnosis and management of pancreatic cancer [3], [4].

miRNAs are naturally occurring, small, single-stranded, noncoding RNAs that mediate gene expression at the post-transcriptional and translational levels in both plants and animals [5], [6]. These molecules play critical roles in human cancers such as pancreatic cancer [7], [8], as well as in cancer behavior, including its proliferation, invasion, migration, apoptosis, and drug resistance. The miRNA functional network of cancer is related to several aspects of tumor pathogenesis [9]. This network may be used to assess the diagnosis and prognosis of cancer as well as to evaluate possible therapeutic options.

miR-26a is a proven tumor suppressor that is significantly downregulated in hepatocellular carcinoma (HCC) [10]. Decreased miR-26a levels have been associated with poor prognosis; these levels are predictive of the therapeutic response of patients with HCC to interferon-α. In addition, miR-26a overexpression has been correlated with significant tumor regression, indicating that miR-26a reintroduction in patients with cancer may be an effective treatment strategy [11]. Our previous study showed that the tumor-specific miR-26a overexpression, driven by a hAFP–TERT dual promoter, decreased the viability of tumor cells in HCC by regulating the expression of the estrogen receptor (ER)-α, progesterone receptor (PR), p53, cyclin D2, and E2 [12]. However, the precise relationship between miR-26a and pancreatic cancer remains unknown.

In the present study, we examined the miR-26a expression levels in human PDAC tissues. We determined the clinical significance of miR-26a downregulation and its roles in cell growth and cell cycle distribution. In addition, we used a murine model to investigate the potential role of miR-26a in pancreatic tumorigenesis. Our findings provide basic information to better understand the pathogenesis of pancreatic cancer and its possible therapeutic strategies.

## Materials and Methods

### Ethics Statement

This study was conducted in accordance with the Helsinki guidelines for human subject studies and was approved by the institutional review board of Second Military Medical University, Shanghai, China. Signed informed consent was obtained from all study participants for sample collection and analysis. All procedures on animals were approved by the Institutional Animal Care and Use Committee of the Second Military Medical University.

### Patients and Tissue Samples

The data on patients with PDAC were retrieved over a 3-year period (January 2008–December 2010) from the Department of Pathology archives of the Changhai Hospital, Second Military Medical University in Shanghai, China and the Shanghai Biobank Network of common human tumor tissue. All patients were treated with surgery, and in total, 106 pairs of samples from PDACs and their ABPT were included. Furthermore, an additional 15 pairs of PDAC and their ABPT samples were collected from patients during surgical resections and stored in liquid nitrogen. The patient characteristics, clinical presentation, staging, laboratory findings, treatment, objective response, survival, and other relevant information were obtained from the hospital information system. Patients were evaluated by standard methods, including their history, physical examination, and biochemical–hematological tests. The TNM Staging System was used to determine the patient disease status.

### Morphological Analysis and Construction of the Tissue Microarray

All specimens were obtained by surgery, fixed in formalin, and embedded in paraffin. Sections of 4-µm thickness were stained with hematoxylin and eosin before being evaluated by three pathologists for the morphological features of pancreatic cancer, according to the World Health Organization classification of the digestive system [13]. Tissue microarrays (TMAs) were constructed as previously described [14]; each microarray contained two panels of 106 PDACs and their ABPT that were arranged on 2.0 mm-diameter cores.

### Reverse-Transcription Reaction and Quantitative Real-Time PCR (qRT-PCR)

In total, 15 pairs of frozen PDACs and their ABPT specimens (macro-dissected) were used for qRT-PCR according to Invitrogen’s protocol. U6 was used as the endogenous control to normalize the quantity of total RNA in each sample. qRT-PCR was performed in triplicate, with nontemplate controls. The relative expression was calculated based on the comparative Ct method (2^−ΔΔ*Ct*^) [12]. The primer sequences are listed in Table S1.

### miRCURY LNA microRNA Detection *in situ* Hybridization and Survival Analysis

Locked nucleic acid (LNA)-*in situ* hybridization (ISH) was performed on PDAC TMA using the respective miRCURY LNA™ probes against has-miR-26a or has-miR-21 (as the positive control) (Exiqon, Vedbaek, Denmark). These probes were used according to the manufacturer’s protocol, as previously described [15]. Colorimetric detection was performed by incubating the samples for 30 min using a substrate–chromogen solution, with 0.04% DAB (DAKO, Denmark) and 0.05% H_2_O_2_. The sections were counterstained with hematoxylin before examination using a light microscope (Leica, Germany) [16].

The LNA-ISH results were semiquantitatively assessed. Based on the intensity of hybridization, samples were scored as “0” for negative; “1” for weakly positive; “2” for moderately positive; and “3” for strongly positive. The percentage of positive epithelial cells was scored as “0” for ≤10%; “1” for 11%–25%; “2” for 25%–50%; and “3” for ≥50%. The intensity and percentage scores were summed to obtain the final ISH score, which was classified as “negative” (<3) or “positive” (≥3) [17].

The LNA-ISH results for miR-26a or miR-21 and the clinical treatment data of 106 patients were further analyzed. The treatment response to chemotherapy and radiotherapy, including the patient’s clinical manifestations,computed tomography (CT), magnetic resonance imaging (MRI) findings and CA19-9 levels, were objectively evaluated. Thus, all the patients included in the study were assessed for their response to treatment, which was rated as “complete response”, “partial response”, “stable disease”, “progressive disease”, “early death from disease or toxicity”, and so on.

### Cyclin D2, Cyclin E2, and PCNA Immunochemistry in Pancreatic Cancer Tissues

The sections were pretreated at 65°C for 2 h, followed by graded deparaffinization. Antigen retrieval was performed prior to incubation with the primary antibodies of cyclin D2 (1∶300 dilution; Millipore), cyclin E2 (1∶300 dilution; Millipore), and proliferating cell nuclear antigen (PCNA) (1∶300 dilution; Dako), overnight at 4°C, with normal IgG as a negative control. Thereafter, slides were incubated for 2 h at room temperature with the HRP-conjugated secondary antibody (1∶100; DAKO). HRP activity was detected using a Liquid DAB+ Substrate–Chromogen System (DAKO). Finally, sections were counterstained with hematoxylin and photographed. The immunohistochemical results were assessed using a semiquantitative method, with the final scores based on the intensity and percentage of positive epithelial cells determined by their immunochemistry; these scores were classified as “negative” (<3) or “positive” (≥3) [17]. The PCNA levels were scored according to the percentage of positive epithelial cells as “0” for ≤5%; “1” for 5%–25%; “2” for 25%–50%; “3” for ≥50%. The samples were classified into groups with a “low proliferative index” (<2) or “high proliferative index” (≥2) [13], [17]. The immunohistochemical results were then further analyzed with follow-up data.

### Cell Culture

The PDAC cell lines Capan-2, SW-1990, and Panc-1, for well (Grade 1), moderately (Grade 2), and poorly (Grade 3) differentiated pancreatic cancer, respectively, were obtained from the Chinese Center for Type Culture Collection (Wuhan, China). Cells were grown in Dulbecco’s minimum essential medium (supplemented with 10% fetal bovine serum) and maintained in a 5% CO_2_ atmosphere at 37°C in an incubator.

### Plasmids and Cell Transfection

The PDAC cell lines Capan-2, SW-1990, and Panc-1 were seeded at 3×10^5^ cells per well in 12-well plates, and transfected with miR-26a mimics, inhibitors, or cyclin E2 siRNA (Dharmacon) at a final concentration of 100 nM using Lipofectamine 2000 (Invitrogen), according to the manufacturer’s protocol.

miR-26a overexpression vector p-hTERT–miR-26a (pTM) and its control vector p-hTERT (pT) were constructed as previously described [12]. To generate stable cell lines, 4×10^5^ cells in each well of a 6-well plate were transfected with 2 µg of plasmids (pTM or pT) using Lipofectamine 2000, according to the manufacturer’s instructions. Positive cultures were selected with 800 µg/ml G418 for 2 weeks.

### Western Blot Analysis

Cells were washed with ice-cold PBS, lysed, and suspended in a lysis buffer (Promega). The resulting total protein extract was separated by 12% SDS-PAGE gels. Immunoblotting was performed on polyvinylidene fluoride membranes. The primary and secondary antibodies used were a rabbit anti-human antibody and a goat anti-rabbit IgG, respectively (Sigma). Immunodetection was performed using an HRP-based chemiluminescent substrate [18]. Table S2 lists the antibodies used in this study.

### Cell Proliferation and Cell Cycle Assays

The proliferative potential of cells was analyzed according to the protocol of the Cell Counting Kit-8 (CCK-8) assay (Dojindo, Japan). The cells were trypsinized, washed twice with PBS, collected by centrifugation, fixed in 70% cold ethanol, incubated with propidium iodide, and analyzed by fluorescence-activated cell sorting (Miltenyi, Germany).

### *In vivo* Tumorigenesis Assay

SW-1990 cells from moderately differentiated PDAC were stably transfected with pTM (p-hTERT–miR-26a) or pT (control vector). The transfected cells were collected, suspended in 200 µl PBS (1×10^7^ cells), and were subcutaneously injected in 4 week-old male BALB/c nude mice (*n* = 8). To avoid the preexisting differences between individual mice, both the stable cell lines (pTM or pT) were individually injected into opposite flanks of the same mouse; cells transfected with pTM were injected into the left flank, whereas the controls (transfected with the vector pT) were injected into the right flank. The mice were maintained in a specific pathogen-free environment for 5 weeks and then sacrificed. The tumor volume *V* was calculated using its length *L* and width *W*, according to the equation *V* = 0.4*LW* ^2^. The mouse xenograft tumors were fixed in formalin and embedded in paraffin wax for the immunochemical analysis of cyclin D2, E2, and PCNA.

### Statistical Analysis

Data were expressed as the mean ± standard deviation (S.D.). The associations of miR-26a expression with the clinical pathologic characteristics were analyzed using the Chi-square or Mann–Whitney tests. The survival curve was constructed using the Kaplan–Meier method and compared using the log-rank test. Groups were compared using a Student’s *t*-test. Correlation analysis was performed using Pearson correlation analysis. All probability values were analyzed using a two-tailed test and considered significant when *P*<0.05. The analyses were performed using SPSS (version 17.0) software (SPSS Inc., Chicago, USA).

## Results

### Downregulation of miR-26a in Pancreatic Cancer Tissues is Associated with Survival

**Figure 1:**
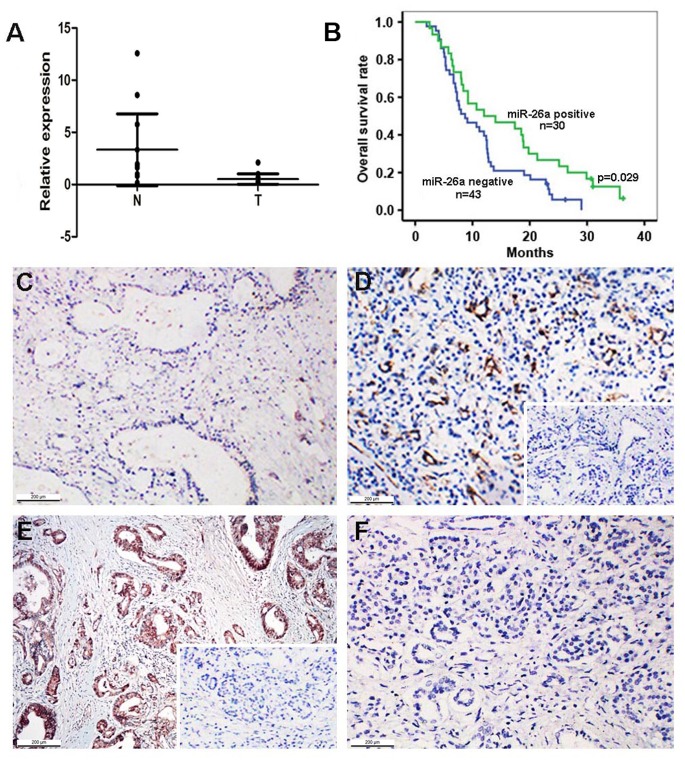
miR-26a expression in pancreatic cancer specimens and overall survival. (A) Average expression level of miR-26a in human PDAC specimens (*n* = 15) and normal pancreatic tissues (*n* = 15). miRNA abundance was assessed by qRT-PCR and normalized to U6 RNA. Values are presented as the mean ± S.D. (B) Overall survival following resection of pancreatic cancer with the miR-26a-negative versus miR-26a-positive groups. The miR-26a-negative group had significantly shorter survival than the miR-26a-positive group (*P* = 0.029). (C,D) *In situ* hybridization for miR-26a in pancreatic lesions. *In situ* hybridization showed much lower miR-26a expression in PDAC tissues (C) than in ABPT (D). The inset shows the negative control (scrambled sequence probe). Cytoplasmic staining in the ductal epithelial cells stands in contrast with the negative staining with the scrambled probe. (E,F) *In situ* hybridization for miR-21 (positive control) in pancreatic lesions. *In situ* hybridization showed much stronger miR-21 expression in PDAC tissues (E) than in ABPT (F). The inset shows the negative control (scrambled sequence probe). Cytoplasmic staining in tumor cells stands in contrast with the negative staining of the scrambled probe. Original magnification, 100×.

The clinicopathological characteristics of 106 cases of PDAC are presented in Table 1. The majority of these patients were in Stage II (70.8%), indicating that the findings are important to patients who are still surgically resectable with the best chance for a 5-year survival. qRT-PCR analysis demonstrated that miR-26a expression was significantly lower in PDAC tissues than in normal tissues (*n = *15, *P*<0.05; Fig. 1A), which was further confirmed by the LNA-ISH analysis of 106 cases of PDAC. LNA-ISH showed that miR-26a was present in the cytoplasm of pancreatic ductal epithelial cells (Figs. 1C and 1D). According to the intensity and percentage scores of the ISH score criteria [17], 44 of the 106 (41.5%) pancreatic cancer tissues and 84 of the 106 (79%) adjacent normal tissues (ISH ≥3; *P*<0.001) were positive for miR-26a. miR-21 expression as the positive control was analyzed in the same patients. miR-21 was positive in 99 of the 106 (93.4%) PDAC tissues and in 25 of the 106 (23.6%) adjacent normal tissues, based on the ISH scoring criteria (Figs. 1E and 1F; ISH ≥3; *P*<0.01). The relationship of clinical characteristics with miR-26a expression in PDAC tissues, as determined by LNA-ISH, is presented in Table 2.

**Table 1:** Clinicopathological characteristics of 106 cases of pancreatic cancer.

		N (%)
Gender		
	Male	70 (66%)
	Female	36 (36%)
Age (years)		
	≤60	54 (50.9%)
	>60	52 (49.1%)
Tumor location in pancreas		
	Head	59 (55.7%)
	Body and tail	47 (44.3%
Tumor size (diameter)		
	≤3.0 cm	23 (21.7%)
	>3.0 cm	83 (78.3%)
Cell differentiation		
	Well	2 (1.9%)
	Moderate	78 (73.6%)
	Poor	26 (24.5%)
Stage		
	I	10 (9.4%)
	II	75 (70.8%)
	III	7 (6.6%)
	IV	14 (13.2%)
Lymph node metastasis		
	Positive	46 (43.4%)
	Negative	60 (56.6%)
Neural invasion		
	Positive	64 (60.4%)
	Negative	42 (39.6%)
Distant metastasis		
	Positive	55 (51.9%)
	Negative	51 (48.1%)

**Table 2:** The relationship between miR-26a expression and clinicopathological features in pancreatic cancer.

			miR-26a expression		
		N (%)	Negative	Positive	P value
Gender					
	Male	70 (66%)	39	31	0.419*
	Female	36 (36%)	23	13	
Age (years)					
	≤60	54 (50.9%)	36	18	0.082*
	>60	52 (49.1%)	26	26	
Tumor location in the pancreas					
	Head	59 (55.7%)	32	27	0.319*
	Body and tail	47 (44.3%)	30	17	
Tumor size (diameter)					
	≤3.0 cm	23 (21.7%)	11	12	0.241*
	>3.0 cm	83 (78.3%)	51	32	
Cell differentiation					
	Well	2 (1.9%)	1	1	0.366**
	Moderate	78 (73.6%)	48	30	
	Poor	26 (24.5%)	13	13	
Stage					
	I	10 (9.4%)	8	2	0.609**
	II	75 (70.8%)	41	34	
	III	7 (6.6%)	5	2	
	IV	14 (13.2%)	8	6	
Lymph node metastasis					
	Positive	46 (43.4%)	29	17	0.591*
	Negative	60 (56.6%)	33	27	
Neural invasion					
	Positive	64 (60.4%)	42	22	0.066*
	Negative	42 (39.6%)	20	22	
Distant metastasis					
	Positive	55 (51.9%)	35	20	0.264*
	Negative	51 (48.1%)	27	24	

* Chi-square test.

** Mann–Whitney test.

Follow-up data obtained from 73 of the 106 patients demonstrated that a total of 18 patients were treated with adjuvant chemotherapy of gemcitabine and oxaliplatin, whereas the remaining 55 patients were not treated with chemotherapy or radiotherapy. Among the 73 patients who received follow-up, 68 died, with 16 patients receiving chemotherapy and 52 without additional therapy. However, 5 of the 73 follow-up patients survived. The measured overall survival was cancer-specific; the median survival time was 8.7 months in the miR-26a-negative group (<3; *n* = 43) and 12 months in the miR-26a-positive group (≥3; *n* = 30). The 1- and 3-year survival rates were 39.5% and 0%, respectively, in the miR-26a-negative group, but were 50% and 6.3%, respectively, in the miR-26a-positive group. The overall survival analysis revealed that the miR-26a-negative group had significantly shorter survival than the positive group (*P* = 0.029, Fig. 1B). Adjuvant chemotherapy was not associated with the survival of patients with PDAC (*P = *0.417). The association between miR-26a expression and other parameters (such as age, gender, tumor mass location, tumor size, tumor cell differentiation, neural invasion, lymph node number, distant metastasis) were not statistically significant (Table 2). The multivariate survival analysis (Cox regression model) of conventional clinical prognostic factors and miR-26a claimed that miR-26a is an independent prognostic factor in pancreatic cancer (*P = *0.001; 95% CI, 0.185 to 0.650; Table 3).

**Table 3:** Mutivariate survival analysis (Cox regression model) of conventional clinical prognostic factors and miR-26a.

Variables	Hazard Ratio	95% Confidence intervals	*P* value
miR-26a	0.335	0.177–0.634	0.001
Age	1.419	0.767–2.624	0.265
Tumor location in the pancreas	1.028	0.510–2.075	0.938
Tumor size	0.380	0.163–0.887	0.025
Cell differentiation	3.275	1.728–6.206	0.000
Neural invasion	1.392	0.705–2.748	0.340
Stage	1.388	0.968–1.992	0.075
Lymph node metastasis	0.669	0.344–1.300	0.236
Distant metastasis	1.352	0.730–2.507	0.338
Adjuvant chemotherapy	1.277	0.670–2.434	0.457

### Cyclin D2, Cyclin E2, and PCNA Immunochemistry in Pancreatic Cancer Tissues and their Association with Survival

**Figure 2:**
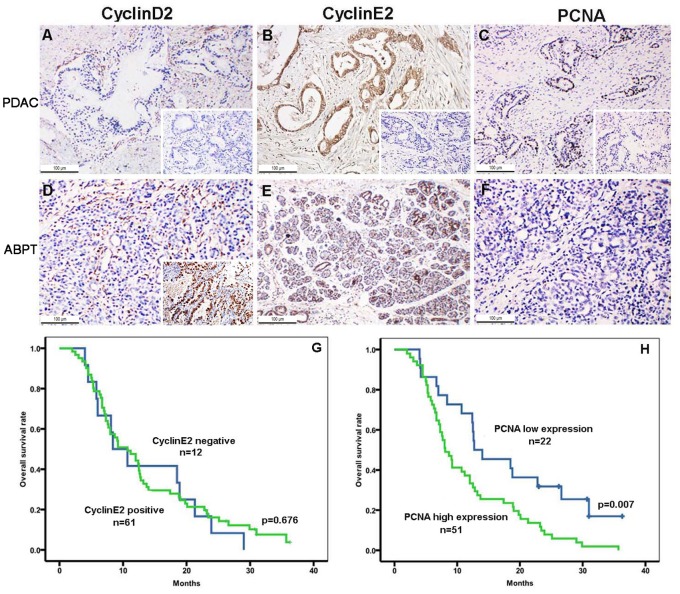
Immunohistochemical expression of cyclin D2, cyclin E2, and PCNA in PDAC tissues as well as overall survival. The PDAC tumor tissues (PDAC) were negative for cyclin D2 (A) and strongly positive for cyclin E2 (B), with a high PCNA proliferative index (C). The adjacent benign pancreatic tissues (ABPT) were negative for cyclin D2 was (D), and positive for cyclin E2, as observed in the ductal epithelial cells of ABPT (E). PCNA was very low in normal pancreatic tissues (F). The insets of A, B, and C show the negative controls. The inset in D indicates that cyclin D2 is a positive control of lung adenocarcinoma. Original magnification, 200×. Overall survival after resection of pancreatic cancer with the cyclinE2-positive versus cyclinE2-negative groups was not significant (*P* = 0.676) (G), whereas overall survival after resection of pancreatic cancer with the PCNA high proliferative index versus PCNA low proliferative index groups had a significantly shorter survival (*P* = 0.007) (H).

The immunostained tissue samples revealed that both the tumor cells and duct epithelial cells of the adjacent benign pancreatic tissues did not express cyclin D2 (Figs. 2A and 2D). However, cyclin E2 displayed strong positive staining in the tumor cells (90/106) and duct epithelial cells of the adjacent benign pancreatic tissues (76/106; *P* = 0.024; Figs. 2B and 2E). There was no significant difference in the overall survival rate between the 73 follow-up patients who were positive (*n = *61) or negative (*n = *12) for cyclin E2 (*P* = 0.676; Fig. 2G). The results presented PCNA-positive staining for tumor cells (93/106) and for duct epithelial cells of the adjacent benign pancreatic tissues (68/106; *P*<0.01). The strong positive staining of PCNA in PDAC tissues indicated a higher cell proliferative activity in pancreatic cancer (Figs. 2C and 2F), as compared with negative staining in adjacent normal tissues. The overall survival analysis revealed that the cases with a PCNA high proliferative index (*n* = 51) in PDAC tissues had significantly shorter survival than the cases with a PCNA low proliferative index (*n* = 22; *P* = 0.007, Fig. 2H). Patients with a high PCNA proliferative index were also cyclin E2 positive.

A correlation analysis for both miR-26a and cyclin E2 expression (Pearson coefficient, *R^2^* = 0.004) and for miR-26a and PCNA expression (*R^2^* = 0.024), showed a significant relationship. The scatter charts of the correlation analysis are presented in Figure S1.

### miR-26a Overexpression Inhibited the Growth of Pancreatic Cancer Cells by the Downregulation of Cyclin E2 and EZH2 Expression

**Figure 3:**
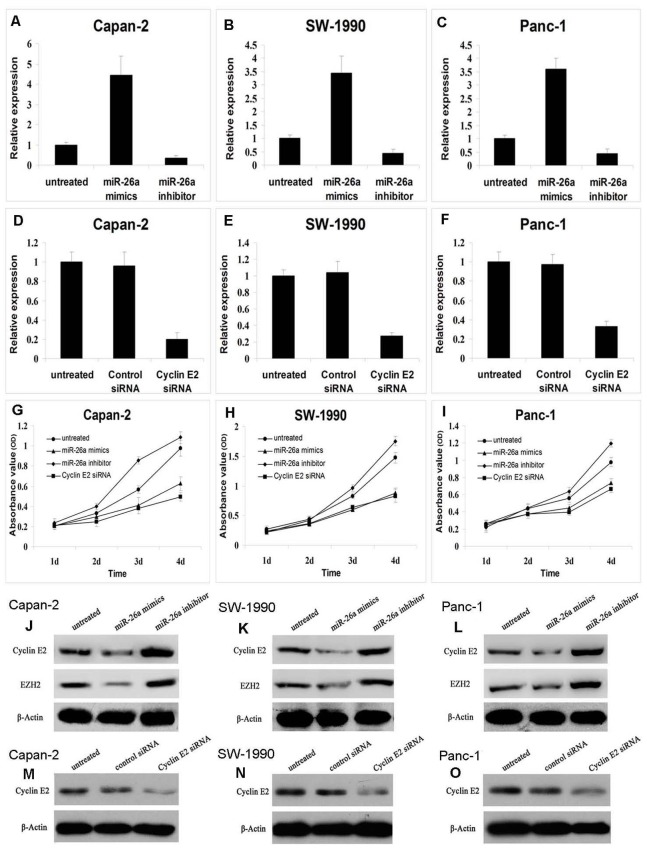
miR-26a overexpression inhibited pancreatic cancer cell growth by the downregulation of cyclin E2 expression. The qRT-PCR analysis demonstrated the transcription of miR-26a in mimics, inhibitor, and control groups (A,B,C), and the expression of cyclin E2 in cyclin E2 siRNA, control siRNA, and control groups (D,E,F). The proliferation of PDAC cell lines transiently transfected with miR-26a mimics, miR26a inhibitor or cyclin E2 siRNA was analyzed by the CCK-8 proliferation assay (G,H,I). The regulation of cyclin E2 or EZH2 expression by miR-26a was analyzed by Western blot (J,K,L), and Western blot analysis also confirmed the expected efficiency of cyclin E2 siRNA in three PDAC cell lines (M,N,O).

To explore the effect of miR-26a on pancreatic cancer cell growth, the PDAC cell lines with different grades Capan-2, SW-1990, and Panc-1 were transiently transfected with a miR-26a mimic or inhibitor. The qRT-PCR analysis showed that the transcription of miR-26a in the mimic group was significantly increased (4.44-fold in Capan-2, 3.45-fold in SW-1990, and 3.59-fold in Panc-1), whereas the transcription of miR-26a in the inhibitor group was significantly decreased (0.35-fold in Capan-2, 0.43-fold in SW-1990, and 0.44-fold in Panc-1), as compared with the control cell lines (*P*<0.05; Figs. 3A–3C).

To explore the effect of cyclin E2 on pancreatic cancer cell growth, cyclin E2 siRNA was transfected into each of the three PDAC cell lines, and the knockdown was confirmed by qRT-PCR analysis, which showed that the expression of cyclin E2 in the siRNA-transfected group was significantly decreased compared with the control cells (0.201-fold in Capan-2, 0.274-fold in SW-1990, and 0.327-fold in Panc-1) (Figs. 3D–3F).

To determine the role of miR-26a in pancreatic cancer cell growth, the CCK-8 proliferation assay was conducted in Capan-2, SW-1990, and Panc-1 cells that were transiently transfected with a miR-26a mimic or inhibitor. The results showed that miR-26a-mimics inhibited tumor cell growth, whereas the miR26a-inhibitor promoted tumor cell growth. In addition, the cyclin E2 siRNA had a similar role as the miR-26a-mimic in inhibiting tumor cell proliferation (Figs. 3G–3I).

miR-26a was reported to directly mediate the cyclin E2 and cyclin D2 functions in HCC [12] and breast cancer [19]. The expression of the polycomb protein EZH2 was increased in PDAC, which consequently increased cell proliferation and chemoresistance [20]. However, our studies showed that cyclin D2 staining was almost negative in pancreatic cancer tissues. Thus, the relationship between miR-26a and cyclin E2 or EZH2 were analyzed using Western blots. The results revealed that cyclin E2 and EZH2 levels were both decreased in the miR-26a mimic-transfected cells, but were increased in the miR-26a inhibitor-transfected cells (Figs. 3J–3L) compared with the control cells. Western blot analysis confirmed the expected efficiency of cyclin E2 siRNA in the three PDAC cell lines (Figs. 3M–3O).

**Figure 4:**
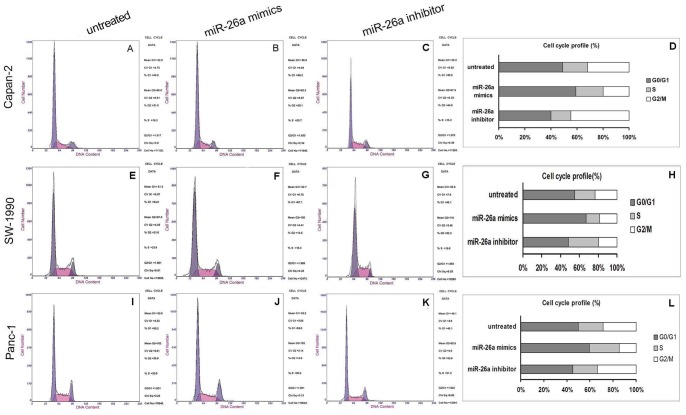
Representation of cell cycle distribution in Capan-2 (A,B,C), SW-1990 (E,F,G), and Panc-1 (I,J,K) cells. Cells were either transfected with miR-26a mimics or inhibitors. The controls were left untreated. D, H, and L indicate the statistical cell cycle distribution for Capan-2, SW-1990, and Panc-1, respectively.

Subsequently, we analyzed the cell cycle distribution of the Capan-2, SW-1990, and Panc-1 cells that were transfected with miR-26a mimics or the miR-26a inhibitor, as well as the controls. The cells transfected with the miR-26a mimic accumulated in the G_1_ phase, whereas the S phase population decreased. However, an opposite result was observed in cells transfected with the miR-26a inhibitor (Fig. 4). These results suggested that the proliferative inhibition of miR-26a was partially due to a G_1_-phase arrest of the three pancreatic cancer cell lines.

### Ectopic Expression of miR-26a Inhibited Pancreatic Cancer Growth in Nude Mice

**Figure 5:**
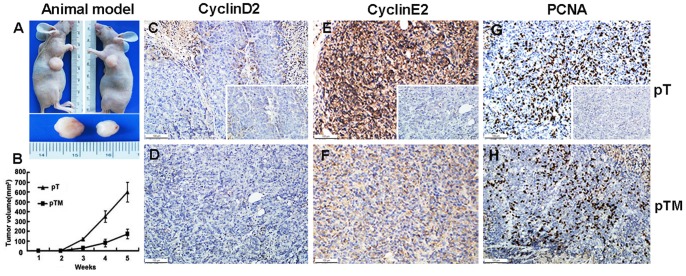
Ectopic expression of miR-26a inhibits pancreatic cancer growth in nude mice. (A) Nude mice were subcutaneously inoculated with SW-1990 cells transfected with pTM (pTM: hTERT–miR-26a plasmid) or pT (pT: hTERT control plasmid), in their flanks. The image is representative of tumors formed in 8 mice. (B) Growth curves of tumor volumes. The graph is representative of tumor growth, 5 weeks after inoculation. Tumor volume was calculated and all data are shown as the mean ± S.D. (*n* = 8). (C–H) Expression of cyclin D2, cyclin E2, and PCNA was measured by immunohistochemistry in the tissues of mice inoculated with pTM- transfected SW-1990 cells or the control cells. The figure insets in C, E, and G indicate a negative control field. Cells colored brown indicate positive staining. Original magnification, 200×.

The *in vivo* tumorigenesis assay revealed that tumor growth was significantly slower in nude mice inoculated with the pTM-transfected SW-1990 cells, as compared with nude mice inoculated with the pT-transfected SW-1990 cells (Fig. 5A). This result was confirmed by the tumor volume measurements at 5 weeks (Fig. 5B). The cyclin E2 level was much lower in tumor tissues overexpressing miR-26a and the PCNA expression followed a similar pattern. Cyclin D2 remained undetected in the tumors (Figs. 5C–5H).

## Discussion

The mature sequence of miR-26a is observed in 3*p*23, which is a fragile chromosomal region associated with various human cancers [21]–[23]. Approximately half of all human miRNAs are located in cancer-associated genomic regions; thus, they can function as tumor-suppressor or oncogenic miRNAs, depending on their targets [3]–[5]. Thus, miR-26a can function as an oncogene in gliomas but serves as a tumor suppressor in liver cancer [12], [23]–[26]. However, to date, there are limited reports on the role and tumorigenesis of miR-26a in pancreatic cancer.

In the present study, we found that the expression of miR-26a was significantly downregulated in PDAC tissues compared with that of ABPT. To further explore the molecular mechanisms of cell growth promotion by miR-26a downregulation in pancreatic cancer tissue, we analyzed the role of miR-26a overexpression or downexpression in pancreatic cancer cell proliferation, cell cycle, and tumor growth,. Our data demonstrated that the expression level of miR-26a influences the proliferation of three PDAC cell lines of different-grades. miR-26a levels were associated with G_1_ arrest in pancreatic cancer cells. In addition, miR-26a overexpression in SW-1990 cells suppressed pancreatic tumorigenesis in nude mice, which suggested that miR-26a functions as a tumor suppressor in this type of cancer.

Cyclin D2 and cyclin E2 are essential regulators of the G_1_-to-S phase transition during the cell cycle. These cyclins are of particular interest in pancreatic cancer. Kota [11] and Zhou [27] reported that miR-26a directly upregulates the expression of cyclin D2 and cyclin E2 mRNA in HCC. Thus, both genes are possible targets of miR-26a in pancreatic cancer. On the other hand, EZH2 levels were increased in PDAC to promote cell proliferation and chemoresistance [20]. Therefore, we determined whether miR-26a could regulate the pancreatic cancer cell cycle through its target genes, cyclin D2 and cyclin E2. Cyclin D2 was not detected in the tumor cells and duct epithelial cells of ABPT, whereas cyclin E2 was positively correlated with miR-26a expression in pancreatic cancer. Cyclin E2 decreased with miR-26a upregulation in Capan-2, SW-1990, and Panc-1 cells. The effects observed on cell proliferation and cyclin E2 expression were consistent with those observed for EZH2 in PDAC cells. These data suggest that miR-26a downregulation led to the upregulation of cyclin E2 and EZH2, but not of cyclin D2, in PDAC tissues. Thus, the downregulated miR-26a contributed to the cell proliferation and poor survival in pancreatic cancer. These results are consistent with studies on other tumors, such as HCC, breast cancer, NPC, and lymphomas [12], [27]–[30]. The altered expression of specific miRNAs in tumors is reportedly associated with cancer metastasis and poor prognosis [29]–[32]. Heinzelmann et al., [33] detected a miRNA signature that distinguishes between metastatic and nonmetastatic clear cell renal cell carcinomas. A group of 12 miRNAs, including the let-7 family, miR-30c, and miR-26a, were found to decrease in highly aggressive primary metastatic tumors. Furthermore, miR-26a expression in primary metastatic clear cell renal cell carcinoma was correlated with patient survival [33].

A single miRNA may actually target several mRNAs, and one mRNA may be regulated by numerous miRNAs. Therefore, the regulatory network of miR-26a is complicated; and other mechanisms could be involved in the miR-26a downregulation of pancreatic cancer progression. Therefore, we propose follow-up studies on the following topics: first, the causes for miR-26a downregulation in pancreatic cancer tissue should be identified. Second, the key regulatory element and translational factor in miR-26a regulation should be elucidated. Third, candidate compounds that upregulate miR-26a need to be screened, especially in pancreatic cancer. Furthermore, the upstream regulatory mechanisms of miR-26a in normal and malignant pancreatic tissues also need to be elucidated.

In summary, we identified miR-26a to be a tumor suppressor miRNA in pancreatic cancer, and low miR-26a expression was an unfavorable prognostic factor in patients with pancreatic cancer. miR-26a partially influences human pancreatic cancer through the regulation of cyclin E2 and EZH2, but not through cyclin D2. These results suggest that miR-26a is a potential target for treating pancreatic cancer and the critical roles of miR-26a in pancreatic cancer tumorigenesis may aid patient prognosis and diagnosis.

## Supporting Information

Figure S1 **Scatter chart of the correlation analysis of miR-26a vs. cyclin E2 expression and the miR-26a vs. PCNA expression.**
(JPG)

Table S1 **Nucleotide sequences in research.**
(DOC)

Table S2 **Antibodies used in immunostaining analysis.**
(DOC)

## References

[1] Hayat MJ, Howlader N, Reichman ME, Edwards BK (2007) Cancer statistics, trends, and multiple primary cancer analyses from the Surveillance, Epidemiology, and End Results (SEER) Program. Oncologist 12(1): 20–37. 17227898 10.1634/theoncologist.12-1-20

[2] Jemal A, Murray T, Samuels A, Ghafoor A, Ward E, et al (2003) Cancer statistics, 2003. CA Cancer J Clin 53(1): 5–26. 12568441 10.3322/canjclin.53.1.5

[3] Delpu Y, Hanoun N, Lulka H, Sicard F, Selves J, et al (2011) Genetic and epigenetic alterations in pancreatic carcinogenesis. Curr Genomics 12(1): 15–24. 21886451 10.2174/138920211794520132 PMC3129039

[4] Jemal A, Siegel R, Ward E, Hao Y, Xu J, et al (2009) Cancer statistics, 2009. CA Cancer J. Clin 59(4): 225–249. 19474385 10.3322/caac.20006

[5] Bartel DP (2004) MicroRNAs: genomics, biogenesis, mechanism, and function. Cell 116(2): 281–297. 14744438 10.1016/s0092-8674(04)00045-5

[6] Croce CM, Calin GA (2005) miRNAs, cancer, and stem cell division. Cell 122(1): 6–7. 16009126 10.1016/j.cell.2005.06.036

[7] Wang J, Sen S (2011) MicroRNA functional network in pancreatic cancer: from biology to biomarkers of disease. J Biosci 36(3): 481–91. 21799259 10.1007/s12038-011-9083-4

[8] Dillhoff M, Liu J, Frankel W, Croce C, Bloomston M (2008) MicroRNA-21 is overexpressed in pancreatic cancer and a potential predictor of survival. J Gastrointest Surg 12(12): 2171–6. 18642050 10.1007/s11605-008-0584-x PMC4055565

[9] Ryu JK, Matthaei H, Dal Molin M, Hong SM, Canto MI, et al (2011) Elevated microRNA miR-21 levels in pancreatic cyst fluid are predictive of mucinous precursor lesions of ductal adenocarcinoma. Pancreatology 11(3): 343–50. 21757972 10.1159/000329183 PMC3142103

[10] Ji J, Shi J, Budhu A, Yu Z, Forgues M, et al (2009) MicroRNA expression, survival, and response to interferon in liver cancer. N Engl J Med 361(15): 1437–47. 19812400 10.1056/NEJMoa0901282 PMC2786938

[11] Kota J, Chivukula RR, O’Donnell KA, Wentzel EA, Montgomery CL, et al (2009) Therapeutic microRNA delivery suppresses tumorigenesis in a murine liver cancer model. Cell 137(6): 1065–17. 10.1016/j.cell.2009.04.021 PMC2722880 19524505

[12] Chen L, Zheng J, Zhang Y, Yang L, Wang J, et al (2011) Tumor-specific expression of microRNA-26a suppresses human hepatocellular carcinoma growth via cyclin-dependent and -independent pathways. Mol Ther 19(8): 1521–8. 21610700 10.1038/mt.2011.64 PMC3149175

[13] Bosman FT, Cameiro F, Hruban RH, Theise ND (2010) World Health Organization Classification of Tumours of the Digestive System. IARC: Lyon.

[14] Sahin F, Maitra A, Argani P, Sato N, Maehara N, et al (2003) Loss of Stk11/Lkb1 expression in pancreatic and biliary neoplasms. Mod Pathol 16(7): 686–91. 12861065 10.1097/01.MP.0000075645.97329.86

[15] Sempere LF, Christensen M, Silahtaroglu A, Bak M, Heath CV, et al (2007) Altered MicroRNA expression confined to specific epithelial cell subpopulations in breast cancer. Cancer Res 67(24): 11612–20. 18089790 10.1158/0008-5472.CAN-07-5019

[16] Jørgensen S, Baker A, Møller S, Nielsen BS (2010) Robust one-day in situ hybridization protocol for detection of microRNAs in paraffin samples using LNA probes. Methods 52(4): 375–81. 20621190 10.1016/j.ymeth.2010.07.002

[17] Moelans CB, Milne AN, Morsink FH, Offerhaus GJ, van Diest PJ (2011) Low frequency of HER2 amplification and overexpression in early onset gastric cancer. Cell Oncol (Dordr) 34(2): 89–95. 21394646 10.1007/s13402-011-0021-0 PMC3063579

[18] Livak KJ, Schmittgen TD (2001) Analysis of relative gene expression data using real-time quantitative PCR and the 2(-Delta Delta C (T)) Method. Methods 25(4): 402–8. 11846609 10.1006/meth.2001.1262

[19] Rachagani S, Kumar S, Batra SK (2010) MicroRNA in pancreatic cancer: pathological, diagnostic and therapeutic implications. Cancer Lett 292(1): 8–16. 20004512 10.1016/j.canlet.2009.11.010 PMC3229224

[20] Avan A, Crea F, Paolicchi E, Funel N, Galvani E, et al (2012) Molecular mechanisms involved in the synergistic interaction of the EZH2 inhibitor 3-deazaneplanocin A with gemcitabine in pancreatic cancer cells. Mol Cancer Ther 11: 1735–1746. 22622284 10.1158/1535-7163.MCT-12-0037 PMC3416916

[21] Chen S, Zheng J, Hao Q, Yang S, Wang J, et al (2010) p53-insensitive PUMA down-regulation is essential in the early phase of liver regeneration after partial hepatectomy in mice. J Hepatol 52(6): 864–71. 20413175 10.1016/j.jhep.2009.12.040

[22] Calin GA, Croce CM (2006) MicroRNA signatures in human cancers. Nat Rev Cancer 6(11): 857–66. 17060945 10.1038/nrc1997

[23] Esquela-Kerscher A, Slack FJ (2006) Incomes-microRNAs with a role in cancer. Nat Rev Cancer 6(4): 259–69. 16557279 10.1038/nrc1840

[24] Calin GA (2004) Human microRNA genes are frequently located at fragile sites and genomic regions involved in cancers. Proc Natl Acad Sci USA 101(9): 2999–3004. 14973191 10.1073/pnas.0307323101 PMC365734

[25] Huse JT, Brennan C, Hambardzumyan D, Wee B, Pena J, et al (2009) The PTEN-regulating microRNA miR-26a is amplified in high-grade glioma and facilitates gliomagenesis in vivo. Genes Dev 23(11): 1327–37. 19487573 10.1101/gad.1777409 PMC2701585

[26] Kim H, Huang W, Jiang X, Pennicooke B, Park PJ, et al (2010) Integrative genome analysis reveals an oncomir/oncogene cluster regulating glioblastoma survivorship. Proc Natl Acad Sci USA 107(5): 2183–8. 20080666 10.1073/pnas.0909896107 PMC2836668

[27] Zhou J, Ju W, Wang D, Wu L, Zhu X, et al (2012) Down-regulation of microRNA-26a promotes mouse hepatocyte proliferation during liver regeneration. PLoS One 7(4): e33577. 22496754 10.1371/journal.pone.0033577 PMC3319545

[28] Zhang B, Liu XX, He JR, Zhou CX, Guo M, et al (2011) Pathologically decreased miR-26a antagonizes apoptosis and facilitates carcinogenesis by targeting MTDH and EZH2 in breast cancer. Carcinogenesis 32(1): 2–9. 20952513 10.1093/carcin/bgq209

[29] Lu J, He ML, Wang L, Chen Y, Liu X, et al (2011) MiR-26a Inhibits Cell Growth and Tumorigenesis of Nasopharyngeal Carcinoma through Repression of EZH2. Cancer Res 71(1): 225–233. 21199804 10.1158/0008-5472.CAN-10-1850

[30] Sander S, Bullinger L, Wirth T (2009) Repressing the repressor: a new mode of MYC action in lymphomagenesis. Cell Cycle 8(4): 556–9. 19197161 10.4161/cc.8.4.7599

[31] Takamizawa J, Konishi H, Yanagisawa K, Tomida S, Osada H, et al (2004) Reduced expression of the let-7 microRNAs in human lung cancers in association with shortened postoperative survival. Cancer Res 64(11): 3753–6. 15172979 10.1158/0008-5472.CAN-04-0637

[32] Ikenaga N, Ohuchida K, Mizumoto K, Yu J, Kayashima T, et al (2010) MicroRNA-203 expression as a new prognostic marker of pancreatic adenocarcinoma. Ann Surg Oncol 17(12): 3120–8. 20652642 10.1245/s10434-010-1188-8

[33] Heinzelmann J, Henning B, Sanjmyatav J, Posorski N, Steiner T, et al (2011) Specific miRNA signatures are associated with metastasis and poor prognosis in clear cell renal cell carcinoma. World J Urol 29(3): 367–73. 21229250 10.1007/s00345-010-0633-4

