# Supplementary figures and images for: The Loss of miR-26a-Mediated Post-Transcriptional Regulation of Cyclin E2 in Pancreatic Cancer Cell Proliferation and Decreased Patient Survival
Source: PLoS One. 2013 Oct 8;8(10):e76450. doi: 10.1371/journal.pone.0076450 (PMC3792981; doi:10.1371/journal.pone.0076450)

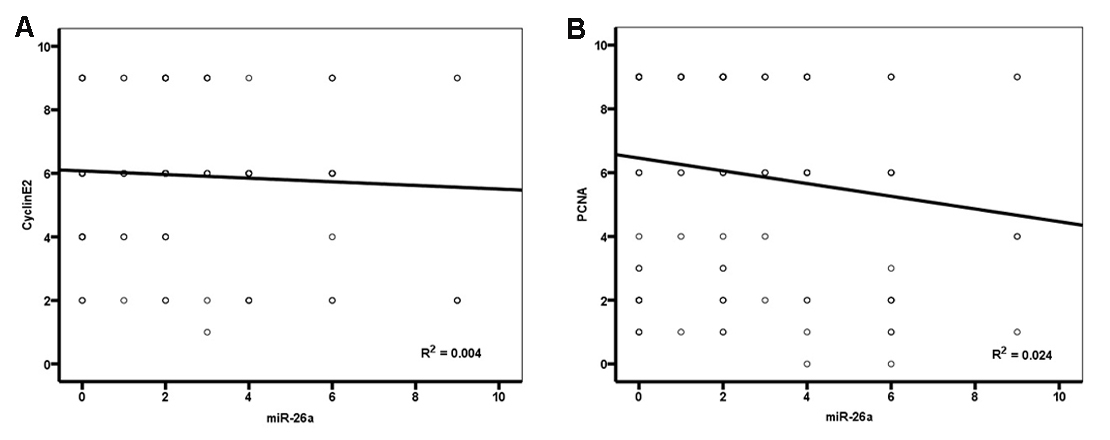

Supplement: Figure S1 — Scatter chart of the correlation analysis of miR-26a vs. cyclin E2 expression and the miR-26a vs. PCNA expression. (JPG) [file pone.0076450.s001.jpg]
