# Supplementary material for: The Loss of miR-26a-Mediated Post-Transcriptional Regulation of Cyclin E2 in Pancreatic Cancer Cell Proliferation and Decreased Patient Survival
Source: PLoS One. 2013 Oct 8;8(10):e76450. doi: 10.1371/journal.pone.0076450 (PMC3792981; doi:10.1371/journal.pone.0076450)
Supplement: Table S1 — Nucleotide sequences in research. (DOC) [file pone.0076450.s002.doc]

|  | **Forward primer sequence (5'-3')** | **Reverse primer sequence (5'-3')** |
| --- | --- | --- |
| U6 for PCR | CTTCGGCAGCACATATAC | GAACGCTTCACGAATTTGC |
| miR-26a for PCR | CTGTCAACGATACGCTAC | GTAATCCAGGATAGGCTG |
| Cyclin E2 for PCR | TCACTGATGGTGCTTGCAGT | GCCAGGAGATGATTGTTACAGGA |
| U6 for RT | GAACGCTTCACGAATTTGC | |
| miR-26a for RT | GCTGTCAACGATACGCTACCTAACGGCATGACAGTGTCAGCCTA | |
| Cyclin E2 siRNA | AACCAAACUUGAGGAAAUCUA | |
| Control siRNA | AACGUACGCGGAAUACUUCGA | |
| has-miR-26a for ISH | GCCTATCCTGGATTA CTTGAA | |
| has-miR-21 for ISH | GCCTATCCTGGATTACTTGAA | |
| hsa-miR-26a mimic | UUCAAGUAAUCCAGGAUAGGCU | |
| hsa-miR-26a inhibitor | AGCCUAUCCUGGAUUACUUGAA | |

**Suppl Table 1. Nucleotide sequences in research**Choose Destination
