# Supplementary material for: The Loss of miR-26a-Mediated Post-Transcriptional Regulation of Cyclin E2 in Pancreatic Cancer Cell Proliferation and Decreased Patient Survival
Source: PLoS One. 2013 Oct 8;8(10):e76450. doi: 10.1371/journal.pone.0076450 (PMC3792981; doi:10.1371/journal.pone.0076450)
Supplement: Table S2 — Antibodies used in immunostaining analysis. (DOC) [file pone.0076450.s003.doc]

**Suppl Table 2. Antibodies used in immunostaining research**

| **Name** | **Host species** | **Molecular weight (KD)** | **Source** |
| --- | --- | --- | --- |
| ACTB | Mouse | 45 | Sigma |
| Cyclin D2 | Mouse | 34 | Millipore |
| Cyclin E2 | Rabbit | 45 | Millipore |
| PCNA | Rabbit | 29 | Dako |
| Ki-67 | Rabbit | 36 | Dako |
| EZH2 | mouse | 98 | Thermo |
| Goat anti-rabbit IgG | | | Abcam |
| Goat anti-mouse IgG | | | Abcam |
